# Applying first & second harmonic spectral phasor analysis on a single-wavelength calcium fluorophore

**DOI:** 10.1016/j.bbrep.2025.101956

**Published:** 2025-02-20

**Authors:** Gabriel Lingotti, Mark R. Jones

**Affiliations:** School of Science, Western Sydney University, Penrith, New South Wales, Australia

## Abstract

Ratiometric and single-wavelength fluorophores are limited in their ability to provide basal level activity prior to treatment. This study presents a novel approach to characterise cell-wide basal activity of calcium using first and second harmonic spectral phasor analysis. Cells stained with the single-wavelength calcium fluorophore Oregon Green™ BAPTA 1-AM exhibited significant differences in wavelength or width in the nucleus, cytoplasm and membrane under basal conditions. Large and small cursor analysis was applied in the first and second harmonic, with smaller cursors revealing a region of interest enveloping and protruding from the nucleus in a structure akin to the sarcoplasmic reticulum. The first harmonic was found to be more sensitive in measurements of *λ_max_*, while the second harmonic showed increased sensitivity in measurements of spectral width. The results of this study indicate that first and second harmonic frequencies should be used in conjunction with phasor analysis of fluorophore microenvironments, rather than the first harmonic alone. Use of this approach may provide more insight into the cellular microenvironment under basal activity and treatment responses.

## Highlights

• · First and second harmonic spectral phasor analysis was used to establish the calcium microenvironment at basal activity.
• · Significant emission differences of a single-wavelength fluorophore was detected across cellular regions.
• · First harmonic showed increased sensitivity in *λ_max_* while the second harmonic was more sensitive in spectral width.
• · Future studies utilising spectral phasor analysis may benefit from using the first and second harmonic together.

## 1 Introduction

Calcium ions play the role of an intracellular messenger, the effects of which are currently detected through fluorescence intensity measurements using either single-wavelength or ratiometric fluorophores [1]. Single-wavelength fluorophores rely on an increase in fluorescence intensity caused by an influx of calcium ions during signalling events, whereas ratiometric fluorophores exhibit significant shifts in excitation or emission during calcium influx [2]. However, both fluorescence detection techniques rely ultimately on an increase in calcium concentration and may not necessarily give detailed information on the cellular microenvironment at basal activity. Further complications arise since single-wavelength fluorophores can introduce artifacts due to uneven dye loading [3], and ratiometric fluorophores require UV excitation wavelengths which can potentially cause phototoxicity and autofluorescence [4].

The phasor approach to fluorescence lifetime imaging (FLIM) has resolved the issues associated conventional FLIM, such as the complexity of resolving multiple decay patterns of the same molecule, providing more information than intensity or ratiometric measurements [5,6]. However, since calcium fluorophores bind to free calcium [3], this presents challenges in characterising the basal activity of the cell as differences in lifetime cannot be determined without differences in calcium binding states. Spectral phasor analysis (SPA) supersedes phasor FLIM and presents a novel method of viewing free calcium at basal level by focusing on the effects of the microenvironment on fluorophore emission, rather than quantifying concentration-dependent binding states [7].

Spectral phasor analysis acquires data through an x, y, *λ* scan. The data undergoes Fourier transformation and the G and S coordinates, which represent the cosine transform and sine transform, respectively, are calculated as below:

(1) $$G_{i}\left(\lambda \right)=\frac{\sum_{\lambda =\lambda_{s}}^{\lambda_{f}}I\left(\lambda \right)\cos\left(n\omega \lambda \right)\Delta \lambda }{\sum_{\lambda =\lambda_{s}}^{\lambda_{f}}I\left(\lambda \right)\Delta \lambda }$$

(2) $$S_{i}\left(\lambda \right)=\frac{\sum_{\lambda =\lambda_{s}}^{\lambda_{f}}I\left(\lambda \right)\sin\left(n\omega \lambda \right)\Delta \lambda }{\sum_{\lambda =\lambda_{s}}^{\lambda_{f}}I\left(\lambda \right)\Delta \lambda }$$

Where *λ*_*S*_ *and λ*_*f*_ *represent the starting and final wavelength of the scan, n is the harmonic number, ω* = 2*πf* with the frequency *f* = (*n* · *spectral channels*)^−1^. Typically, 32 channels are used for x, y, *λ* scans and the calculations for G and S are then used to generate the phasor plot as shown below:

(3) $$phasorplot\left(G,S\right)=\left\{ \begin{array}{c} \sum_{i}hist\left(G_{i}\left(\lambda \right)\right)\\ \sum_{i}hist\left(S_{i}\left(\lambda \right)\right) \end{array}\right.$$

Along with the phasor plot, a spectral image is also generated. Each pixel in the phasor plot contains a spectral profile consisting of the maximum wavelength (*λ_max_*) and full width of the half maximum (spectral width). The former is plotted in an angular fashion while the latter is plotted increasingly to the origin of the phasor plot in a radial fashion.

Cursors are applied to the phasor plot to select clusters of pixels and characterise the average *λ_max_* and spectral width, which is a function of the individual user, cursor placement and size in relation to the G and S values of the phasor plot. Along with the phasor plot, the spectral image directly corresponds to the cursor size and colour, allowing for both a quantitative and qualitative comparison of fluorophore activity across cellular regions.

Spectral phasor analysis can be achieved at a harmonic number n, as seen in equations (1), (2)). Analysis using the first harmonic is most common, and this has been applied to the characterisation of fluorophores in RNA, sodium and lipid membrane microenvironments [[8], [9], [10]]. While characterisation using the second harmonic is possible, its usage on live cells is currently limited. However, the successful implementation of the second harmonic on photo-activatable fluorescent proteins suggest its potential application on live cells [11]. The second harmonic differs from that of the first in that the spectrum acquired through the x, y, *λ* scan is plotted over 4π radians, as opposed to 3/4π radians in the first harmonic, which results in a more distributed and linear position of the dataset in the phasor plot [11]. Additionally, any differences seen in the spectrum are amplified via the shorter periods of sine and cosine in equations (1), (2)). This may ultimately provide an increased sensitivity compared to the first harmonic and may expand the scope of measurement of fluorophore emission changes when both harmonic frequencies are used together.

This present study aims to use first and second harmonic SPA for the detection of emission differences of the single-wavelength fluorophore Oregon Green™ BAPTA 1-AM (OGB) across live cells in basal conditions. Visible light excitable, single-wavelength fluorophores such as OGB are more manageable in the context of their effects of phototoxicity and autofluorescence [12]. Using SPA to characterise this fluorophore may present a new method of analysing calcium activity across live cells in a manner that is independent of fluorophore concentration which intensity measurements are usually dependent on. This study compared the ability of first and second harmonic SPA to detect emission differences across the cell including the nucleus, cytoplasm and membrane, as well as other potential organelles such as the sarcoplasmic reticulum (SR). It is suggested that using the first and second harmonic in conjunction may further the understanding on the effects of different cellular microenvironments on fluorophore dynamics at basal conditions in ways that may affect future studies under treatment conditions.

## 2 Method

### 2.1 Cell culture

The adherent cell line L6 skeletal myoblasts were cultured in Dulbecco's Modified Eagle's Medium (DMEM) and 10 % Foetal Bovine Serum (FBS) and were incubated at 37 °C and 5 % CO2. Cells were seeded on a glass-bottomed dish for imaging.

### 2.2 Calcium staining

Prior to staining, the medium was replaced with 1 ml of 1X phosphate buffered saline (PBS). Cells were stained with 1 nmol of the single wavelength fluorophore OGB. Cells were then incubated for 30 min at 37 °C and 5 % CO2.

### 2.3 Data acquisition

Data acquisition was undertaken through the Leica TCS SP5 inverted confocal microscope using the HC PL APO CS2 63 x 1.2 water objective. A resolution of 256 x 256 was used for all images and scans at 12 bits. All data was obtained using the Argon laser, with the excitation wavelength set at 488 nm at 30 % maximum laser power. For fluorescence data, two fluorescence detection channels were set at 494–629 nm and 639–799 nm along with a pseudo-brightfield channel set at 633 nm. All fluorescence images were taken at 10Hz. For spectral data, an x,y,*λ* scan was set utilising a detection bandwidth range of 496–792 nm, a 100Hz scan speed, a 9.7 nm bandwidth and 32 detection steps. During acquisition of both images and spectral scans, the focal plane was focused on the medial aspect of the cells.

### 2.4 Spectral phasor analysis

Spectral phasor analysis was performed using Global Images software SimFCS 4.0 (Laboratory for Fluorescence Dynamics, University of California, Irvine). The spectral data was referenced and read into SimFCS (S1 fig). Background was removed up to the cell membrane using the masking tool, as shown in Figs. S2 and S3.

Three cursors were assigned a size of 0.05 (large) and were used to select clusters of pixels in the nucleus, cytoplasm and membrane, pseudo-colouring them red, blue and green, respectively, according to the cursor colour. Four cursors were assigned a size of 0.005 (small) and used to characterise the nucleus, SR-like ROI, cytoplasm and membrane and pseudo-coloured the pixels as red, blue, green and yellow, respectively.

### 2.5 Statistical analysis

Both *λ_max_* and spectral width values obtained through SimFCS were transferred into GraphPad Prism 9 for statistical and graphical representation. All data is presented as a column graph of the mean ± SEM. Statistical analysis was performed using the Repeated Measures ANOVA test.

## 3 Results and discussion

### 3.1 First and second harmonic large cursor analysis

Firstly, a fluorescent image was taken at 10Hz (Fig. 1A) of skeletal myoblasts stained with OGB. While the nucleus can be visually distinguished from the cytoplasm and membrane, this image provides little detail on the cellular microenvironment. Calcium signalling events are typically characterised through significant increases in concentration [2]. This leads to either increases in fluorescence intensity in single-wavelength fluorophores or significant changes in the excitation/emission of ratiometric fluorophores [3]. Whilst the influx of calcium occurs across the cell, including the nucleus, calcium activity mostly occurs in the cytoplasm [13]. Small changes in the emission of the fluorophore are, however, independent from fluorophore concentration and may therefore be more indicative of the cellular microenvironment. SPA presents a new method to detect calcium activity in cells, via differences in the emission of a fluorophore associated with different microenvironments. This method has been demonstrated to be independent of fluorophore concentration and can therefore be applied to characterise live cells at both basal level and following a stimulus.

**Fig. 1:**
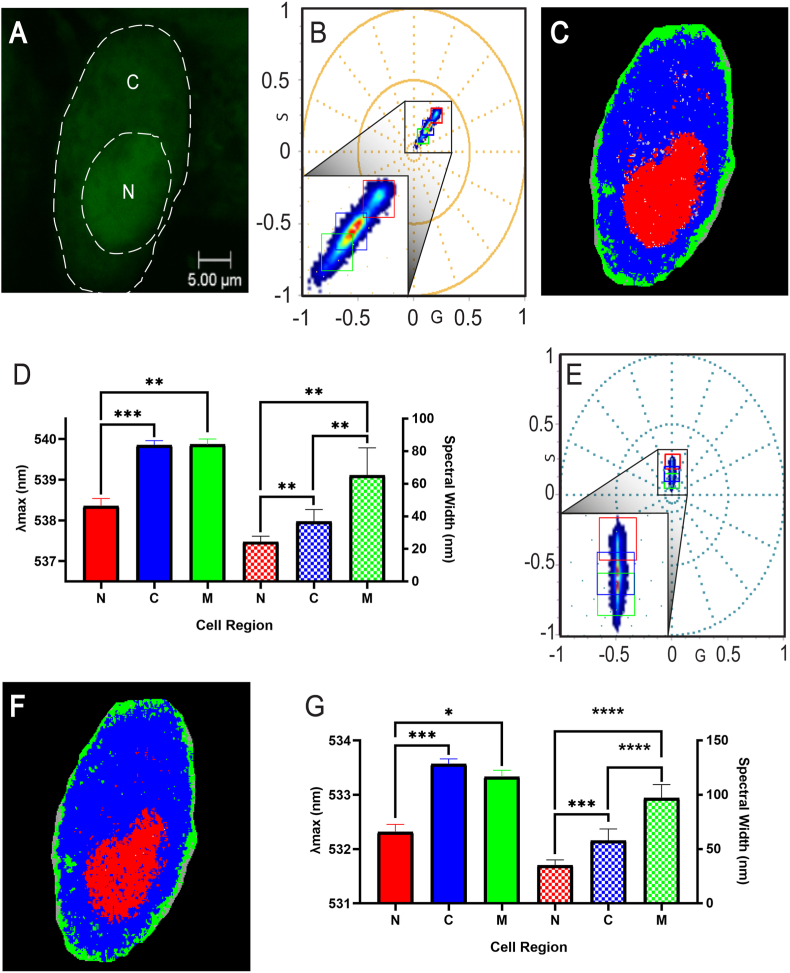
**First and Second Harmonic Spectral Properties of OGB in three cellular regions using large cursors (0.05)**. **(A)** Fluorescence image with a 5 μm scale bar, as well as labels for the cytoplasm (C) and nucleus (N). **(B)** first harmonic spectral phasor plot with a zoomed image showing all three cursors outlining the nucleus (red), cytoplasm (blue) and membrane (green). **(C)** spectral image that corresponds with the first harmonic phasor plot and each cursor applied. **(D)**. Average first harmonic *λ_max_* (left y-axis) and spectral width (right y-axis) of the nucleus (N), cytoplasm (C) and membrane (M) of all cells analysed. **(D)** Second harmonic spectral phasor plot with a zoomed image showing all three cursors outlining the nucleus (red), cytoplasm (blue) and membrane (green). **(E)** Spectral image that corresponds with the second harmonic phasor plot and each cursor applied. **(F)** Spectral image that corresponds with the second harmonic phasor plot and each cursor applied. **(G)** Average second harmonic *λ_max_* (left y-axis) and spectral width (right y-axis) of the nucleus (N), cytoplasm (C) and membrane (M) of all cells analysed.

Large cursors (0.05) in the first harmonic were used to isolate the emission profiles of OGB across live cell including the nucleus, cytoplasm and membrane (Fig. 1B and C). The average *λ_max_* of the nucleus was 538.4 nm ± 0.2 nm, which was lower than that of both the cytoplasm (539.9 nm ± 0.1 nm) and membrane (539.9 nm ± 0.1 nm). Statistical analysis using the repeated measures ANOVA indicated that the nucleus yielded significant differences to the cytoplasm (p < 0.001) and membrane (p < 0.01). The spectral resolution shown between the nucleus and cytoplasm/membrane (<2 nm) has been shown in both simulations and live cells analysis [8,14]. Golfetto et al. demonstrated a spectral resolution of up to 2 nm in simulations of 10 overlapping spectra, while Pyronin-Y labelled RNA species in live cells revealed a sensitivity of <5 nm [8,14]. Spectral differences between the cytoplasm and nucleus can occur, with cytoplasmic and nucleoplasmic regions being shown to display different spectral qualities [15]. Long-wavelength fluorophores, such as OGB, negate autofluorescence when considering spectral shifts [12]. The results presented here demonstrate the presence of microenvironment-related differences in OGB emission between the nucleus and cytoplasm/membrane.

Spectral width analysis in the first harmonic yielded an average of 24.3 nm ± 1.5 nm in the nucleus, which was lower than that of the cytoplasm (36.9 nm ± 3 nm) and membrane (65.2 nm ± 6.9 nm). Significant differences of spectral width values were observed between each cellular region (Fig. 1D). Whilst the cytoplasm and membrane displayed identical *λ_max_* values, spectral width values contained differences of >25 nm, showcasing the advantage of the phasor approach in distinguishing emission profiles with overlapping *λ_max_* values [14]. Use of the first harmonic enabled the discernment of differences in fluorophore activity across the nucleus, cytoplasm and membrane in basal activity via differences in *λ_max_* and/or spectral width.

Second harmonic analysis (Fig. 1E and F) isolated the emission profiles of OGB in the nucleus, cytoplasm and membrane with similar precision to that of the first harmonic. The nucleus displayed an average *λ_max_* of 532.3 nm ± 0.1 nm, which was significantly lower than that of the cytoplasm (533.6 nm ± 0.1 nm, p < 0.001) and the membrane (533.3 nm ± 0.1 nm, p < 0.05). The average spectral width of the nucleus was 35.1 nm ± 2 nm, while the cytoplasm and membrane contained an average width of 57.9 nm ± 4.4 nm and 97.0 nm ± 5.1 nm, respectively. Much like the first harmonic, significant differences were observed between each cellular region (Fig. 1G).

Interestingly, Fig. 1D and G shows that while the first harmonic displayed more sensitivity in *λ_max_* analysis, the second harmonic was more sensitive towards spectral width. The second harmonic phasor plot is located closer toward the origin and since spectral width is plotted increasingly towards the origin, this is likely the reason towards the increased sensitivity. In theory, the second harmonic should be more sensitive in both *λ_max_* and spectral width as the shorter periods of sine and cosine in equations (1), (2)) are amplified more than in the first harmonic [11]. However, it should be noted that this increased sensitivity of the second harmonic may make it more sensitive to noise [11], which may impact the ability to detect differences in *λ_max_*. This sensitivity is a result of the extra wrap around effect, which is 4/4π quadrants as opposed to 3/4π quadrants in the first harmonic [14]. In contrast, the first harmonic is less sensitive to noise, which is due to the smaller wrap around effect as well as it having the highest amplitude [7,14]. The results of this study highlight how measurements using both the first and second harmonic each have their advantages and disadvantages. It is suggested here that future studies of fluorophore dynamics in live cells would greatly benefit from using both harmonic frequencies for analysis, rather than the first harmonic alone.

Clusters of pixels, identified by each of the assigned cursor size and colour, contain an average value of both *λ_max_* and spectral width across all pixels selected. The application of SPA may have limitations in regard to overlapping of cursors, which leads to overlapping of the emission profiles of the pixel groups and impacts the spectral resolution of subcellular regions. Linking cursors, as described in its application of sodium microenvironments [9], enables the use of overlapping cursors when characterising cellular fluorophore activity, but not subcellular activity. Reducing the cursors size may enable the selection of smaller clusters of pixels and reduce the overlapping of regional emission profiles, making it better suited in the analysis of subcellular components.

### 3.2 First and second harmonic small cursor analysis

The cytoplasm of cells is considered to be a highly crowded medium, containing proteins, macromolecules and organelles [16]. The SR is an organelle that plays a vital role in the release and reuptake of intracellular calcium ions, necessary for calcium signalling events seen in muscle contraction [17]. The SR is highly heterogeneous and may therefore contain its own unique calcium microenvironment compared to the rest of the cell [18]. Reducing the assigned cursor size enables the determination of microenvironments within regions of the cell. In contrast, large cursors contain issues in the overlapping of pixels and their emission profiles across cellular components. Consequently, 4 small cursors (0.005) were applied in order to determine whether SR-like regions of interest (ROIs), along with the nucleus, membrane and other cytoplasmic ROIs, could be observed in live skeletal myoblasts stained with OGB.

### 3.3 First harmonic

Application of 4 small cursors (0.005) in the first harmonic identified 4 distinct regions across the cell, with no overlapping cursors observed (Fig. 2). The nucleus, highlighted by the red cursor, contained an average *λ_max_* of 538.3 nm ± 0.2 nm and spectral width of 25.4 nm ± 1.7 nm. Significant differences were of both *λ_max_* and spectral width were observed between each cellular region (Fig. 2H). An SR-like ROI was observed, highlighted by the blue cursor, which envelopes and protrudes from the cell nucleus (Fig. 2C). These results agree with the literature, as the SR is known to be continuous with the nuclear membrane [18]. This region produced an average *λ_max_* and spectral width of 540.0 nm ± 0.3 nm and 30.8 nm ± 2.6 nm, respectively. A significant difference of *λ_max_* was observed between the nucleus (p < 0.01), while significant differences in width were observed between the nucleus and cytoplasm (p < 0,05). Surrounding the SR-like ROI is the rest of the cytoplasmic region (Fig. 2D). The cytoplasm is likely to contain its own unique microenvironment and encompasses other organelles such as mitochondria, which itself serves as a regulator of intracellular calcium [19]. The cursor highlighting the cytoplasm, coloured green, displayed an average *λ_max_* and spectral width of 539.8 nm ± 0.3 nm and 38.2 ± 4.4 nm. The membrane, highlighted in yellow, is known to contain membrane-bound transporter proteins that interact with the regulation of intracellular calcium. However, *λ_max_* (539.7 nm ± 0.2 nm) and spectral width (55.4 nm ± 9.0 nm) of this region yielded no significant differences between the cytoplasm and SR-like ROI. While significant differences in *λ_max_* and width were observed when compared to the nucleus (Fig. 2H), these results suggest that the first harmonic was not able to identify membrane-related microenvironments separate from the cytoplasm and SR.

**Fig. 2:**
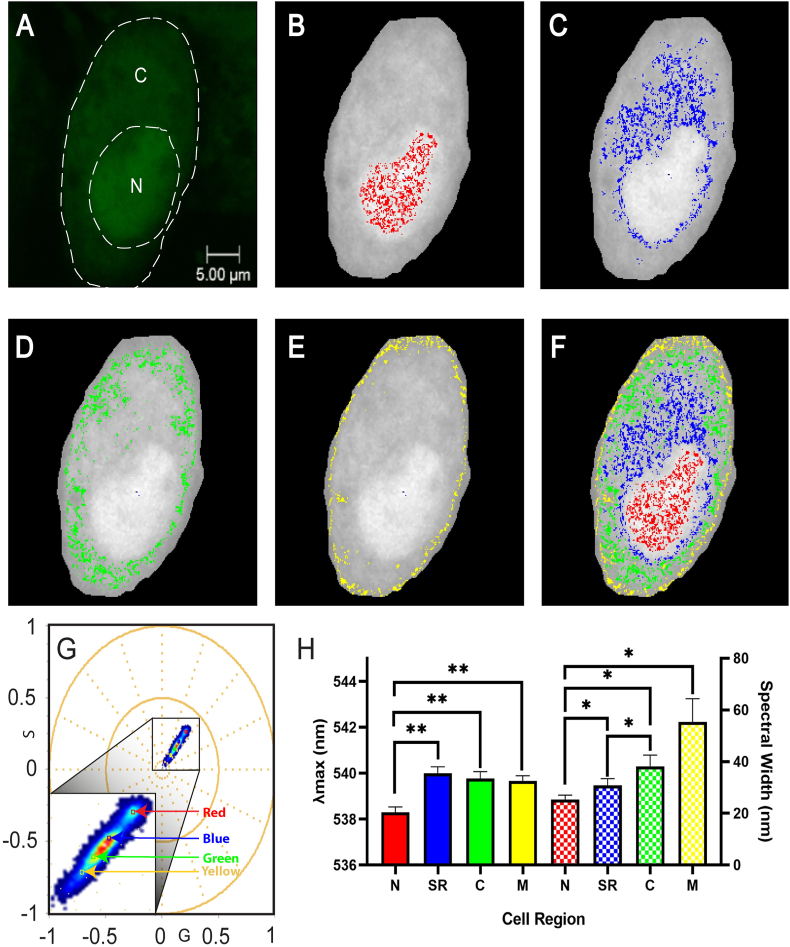
**First Harmonic Spectral Properties of OGB in four cellular regions using small cursors (0.005)**. **(A)** Fluorescence image with a 5 μm scale bar, as well as labels for the cytoplasm (C) and nucleus (N). Individual spectral images were generated which highlight each region across the cell such as the **(B)** nucleus, **(C)** SR-like ROI enveloping and protruding from the nucleus, **(D)** cytoplasmic ROIs and **(E)** cell membrane. **(F)** An overlay of each cellular region highlighted by the cursors in **(G)** the spectral phasor plot was generated. **(H)** Average first harmonic *λ_max_* (left y-axis) and spectral width (right y-axis) of the nucleus (N), SR-like ROI (SR), cytoplasm (C) and membrane (M) of all cells analysed. Data is expressed as a column bar of the mean and SEM. Statistical analysis was conducted using the Repeated Measures ANOVA.

### 3.4 Second harmonic

Second harmonic analysis was also able to accurately identify the 4 different regions across the cell and, much like the first harmonic, contained no overlapping cursors (Fig. 3). The nucleus, shown in Fig. 3B, exhibited an average *λ_max_* of 532.8 nm ± 0.1 nm, which was significantly lower than the SR-like ROI (533.8 nm ± 0.2 nm, p < 0.05) and the membrane (533.5 nm ± 0.2 nm, p < 0.05). The cytoplasm yielded an average *λ_max_* of 533.4 nm ± 0.1 nm, which showed no significant differences between those of other cellular regions. These results provide further evidence that although the second harmonic exhibits reduced sensitivity in the detection of *λ_max_* differences than that of the first harmonic, second harmonic spectral width analysis indicated increased sensitivity. Indeed, the nucleus exhibited an average spectral width of 37.3 nm ± 2.4 nm, which was lower than the SR-like ROI (46.1 nm ± 3.8 nm), cytoplasm (61.4 nm ± 6.4 nm) and membrane (90.0 nm ± 13.6 nm). In contrast to the first harmonic, each cellular region contained significantly different spectral width values (Fig. 3H). As such, the results of this study provide evidence that the first harmonic can detect differences in fluorophore emission not detected by the second harmonic and vice-versa. This suggests that analysis of the cellular microenvironment should be undertaken by both harmonic frequencies used together, rather than the first harmonic alone.

**Fig. 3:**
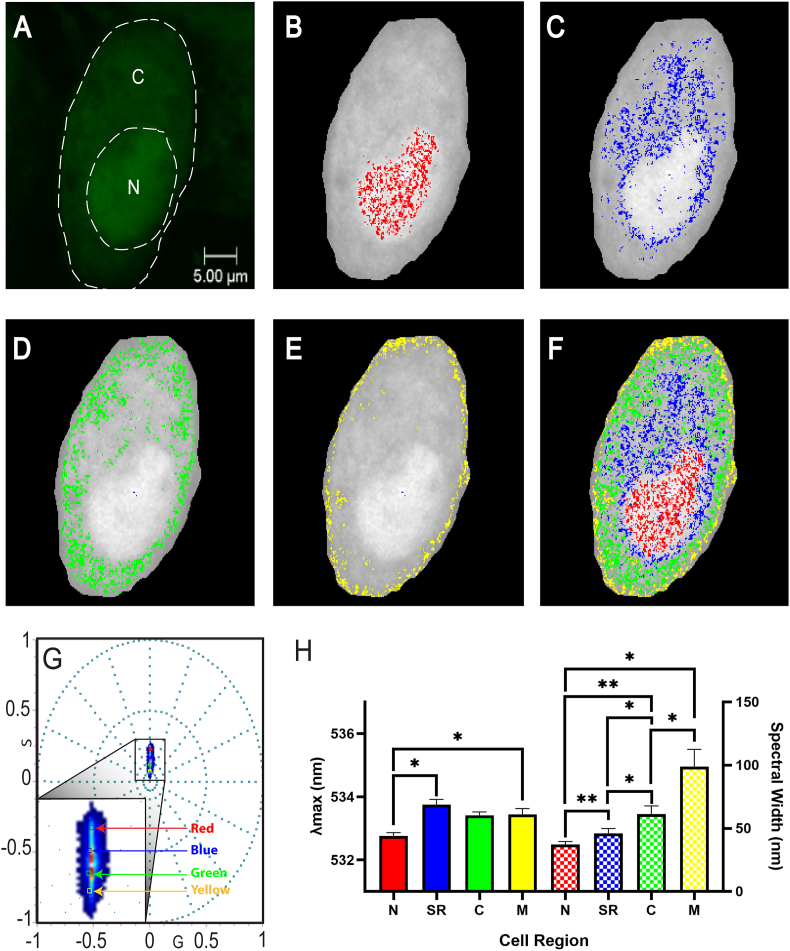
**Second Harmonic Spectral Properties of OGB in four cellular regions using small cursors (0.005)**. **(A)** Fluorescence image with a 5 μm scale bar, as well as labels for the cytoplasm (C) and nucleus (N). Individual spectral images were generated which highlight each region across the cell such as the **(B)** nucleus, **(C)** SR-like ROI enveloping and protruding from the nucleus, **(D)** cytoplasmic ROIs and **(E)** cell membrane. **(F)** An overlay of each cellular region highlighted by the cursors in **(G)** the spectral phasor plot was generated. **(H)** Average first harmonic *λ_max_* (left y-axis) and spectral width (right y-axis) of the nucleus (N), SR-like ROI (SR), cytoplasm (C) and membrane (M) of all cells analysed. Data is expressed as a column bar of the mean and SEM. Statistical analysis was conducted using the Repeated Measures ANOVA.

The use of SPA establishes the basal activity of the calcium microenvironment to enable an understanding of the alterations of the cell in future studies, including calcium signalling events across different cellular regions [18]. Using SPA enables the separation of multiple fluorophores in a sample [7], pointing towards future research utilising co-staining between calcium dyes and other fluorophores to enable analysis in specific regions of the cell. This study utilised a wavelength range of 296 nm (496–792 nm) in the x, y, *λ* scan, which has limitations in the context of scan time (104.83 s). The results presented here showed differences in *λ_max_* and spectral width of <2 nm and >50 nm, respectively, which suggests shortening the wavelength range to 50–100 nm may be possible to address issues with scan time. This may be vital in future studies utilising a stimulus, as calcium signals can be generated in a matter of seconds.

## 4 Conclusion

This study sought to determine whether first and second harmonic SPA can detect emission differences in the single wavelength fluorophore OGB across live cells at basal activity. Wavelength or width differences were detected across the cell in both the first and second harmonic using large cursors, although overlapping of cursors was evident. This was circumvented by using small cursors, which also identified four subcellular spaces, including an SR-like ROI around the nucleus. However, future studies using a fluorophore targeting the SR may be required in order to confirm this. The results of this study also demonstrated that the first harmonic was more sensitive towards differences in *λ_max_*, while the second harmonic contained increased sensitivity in spectral width differences. This suggests that future studies characterising the cellular microenvironment may benefit from using both harmonic frequencies together, rather than the first harmonic alone. The use of this approach has established the calcium microenvironment across cellular components at basal activity, allowing for future analysis of stimuli-induced alterations.

## CRediT authorship contribution statement

**Gabriel Lingotti:** Writing – original draft, Methodology, Investigation, Formal analysis, Data curation, Conceptualization. **Mark R. Jones:** Writing – review & editing, Supervision, Methodology.

## Funding sources

All funding from 10.13039/501100001776University of Western Sydney.

## Declaration of competing interest

The authors declare that they have no known competing financial interests or personal relationships that could have appeared to influence the work reported in this paper.

## Figures and Tables

**figs1:**
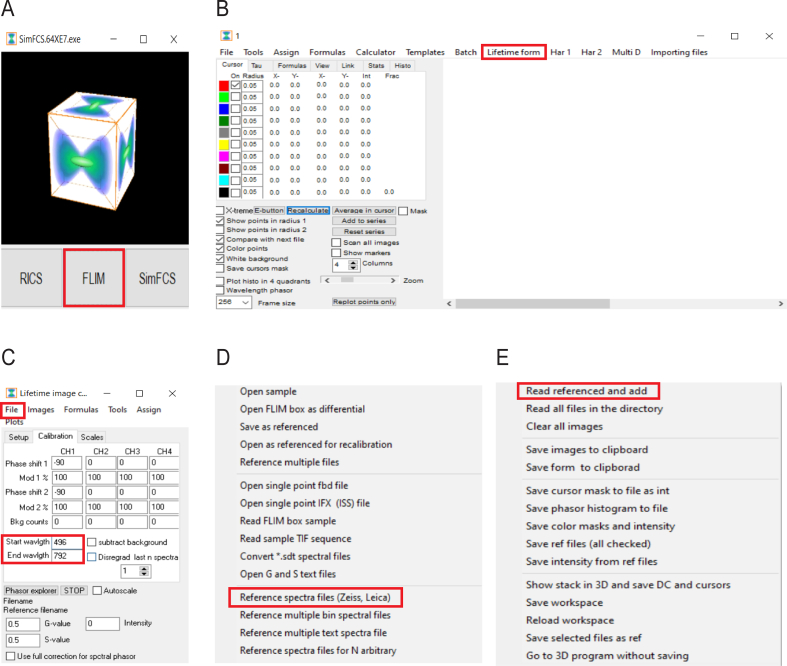


**figs2:**
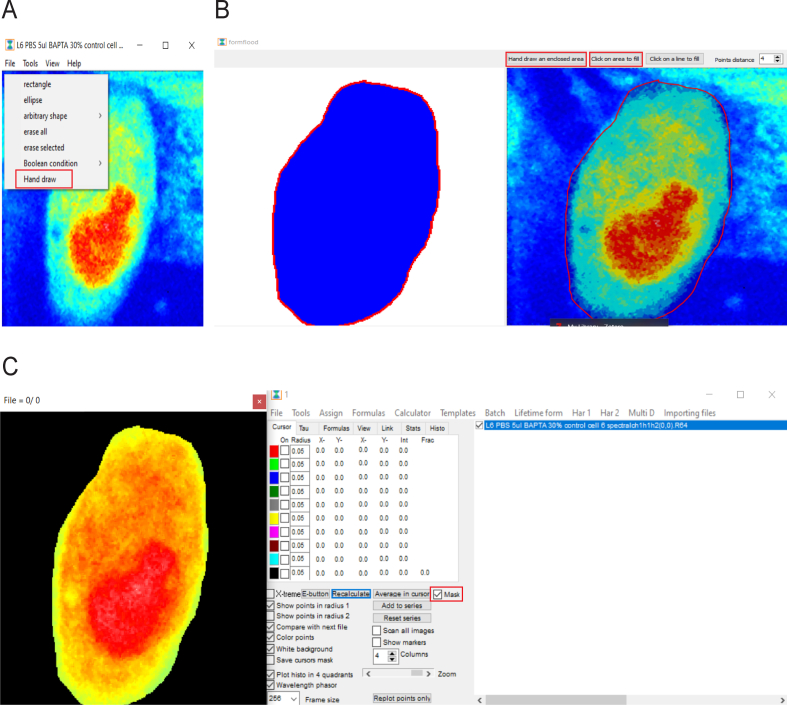


**figs3:**
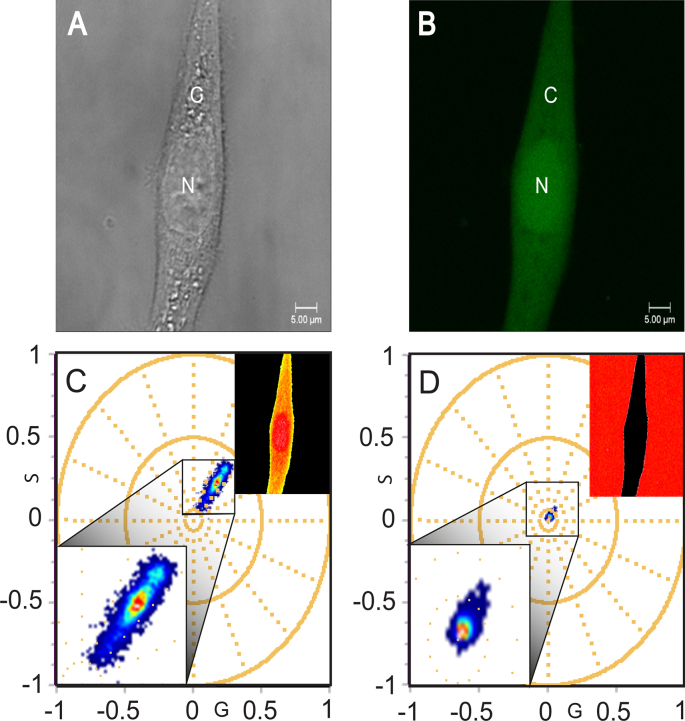


**figs4:**
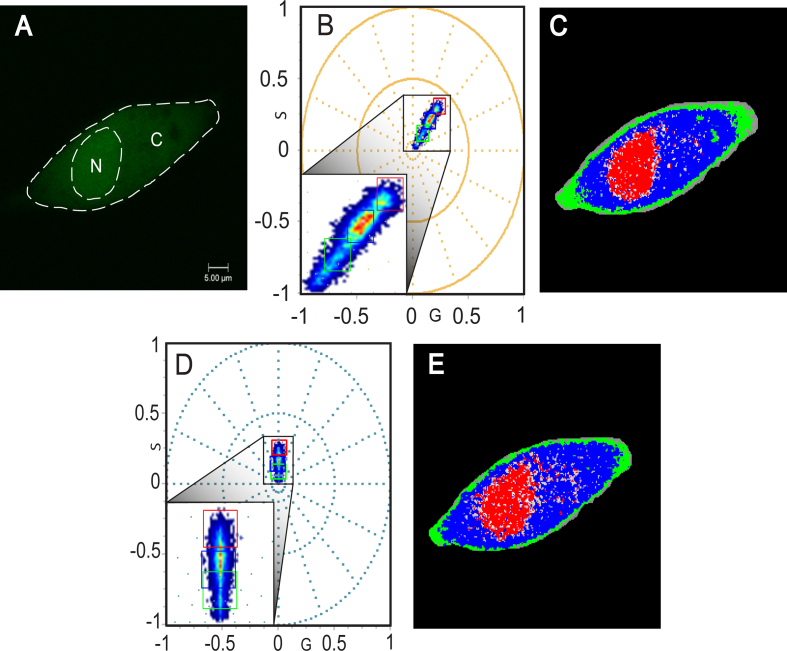


**figs5:**
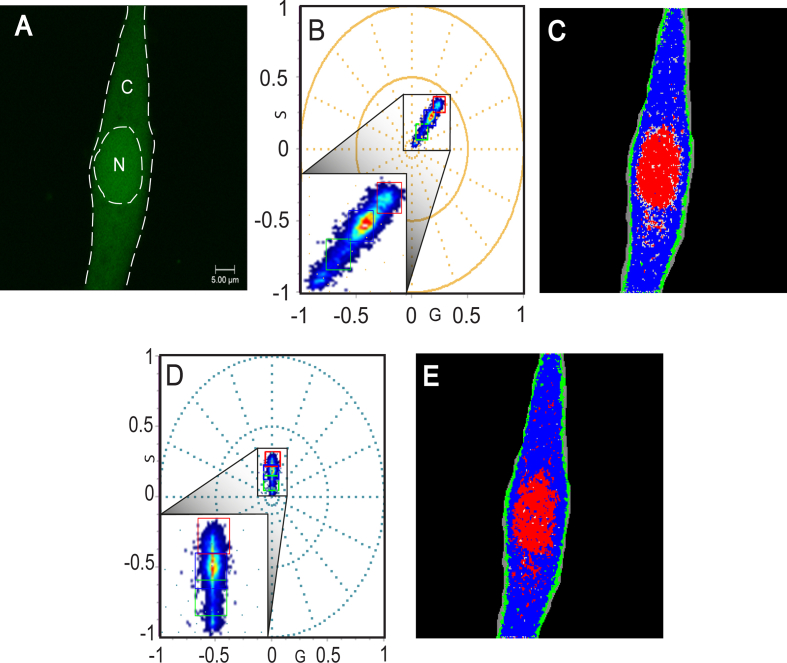


**figs6:**
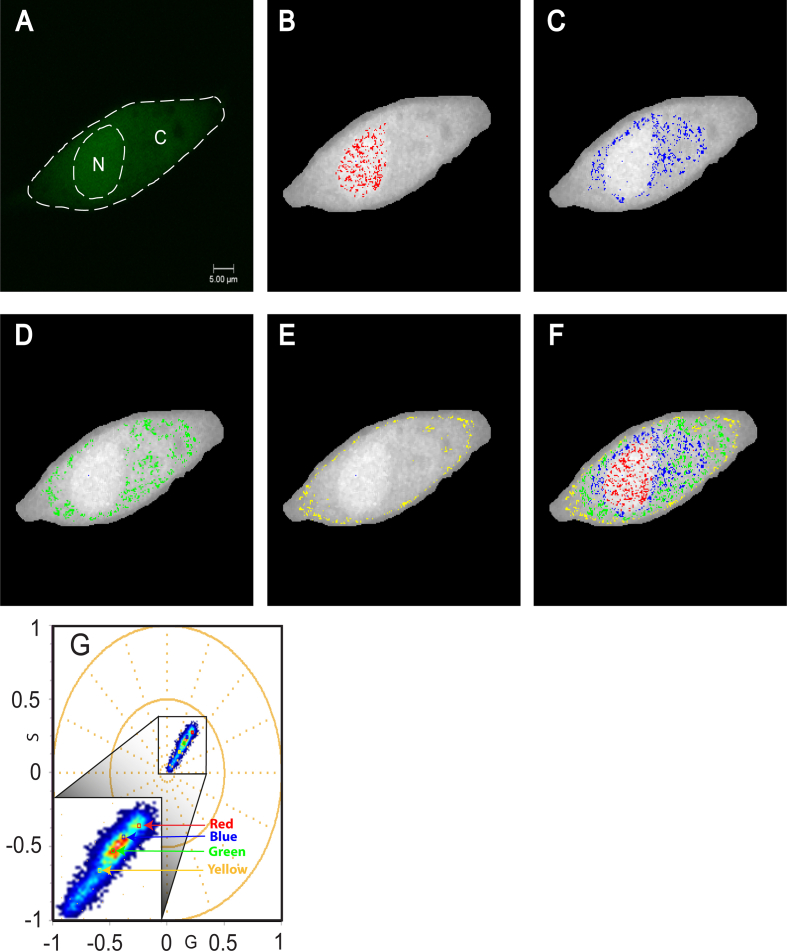


**figs7:**
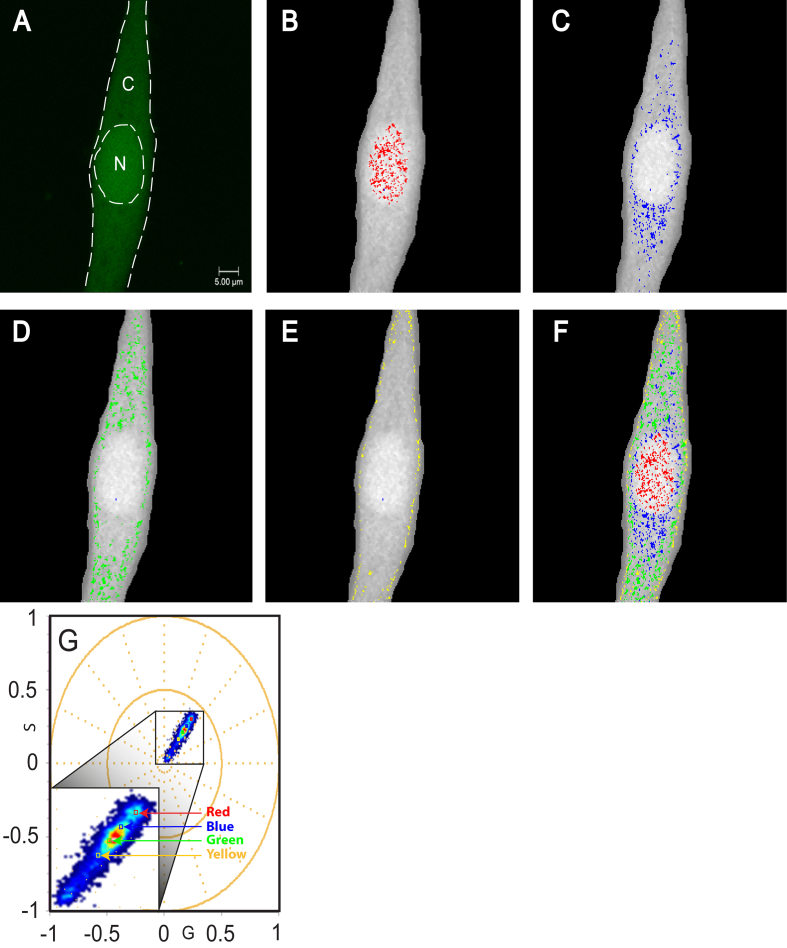


**figs8:**
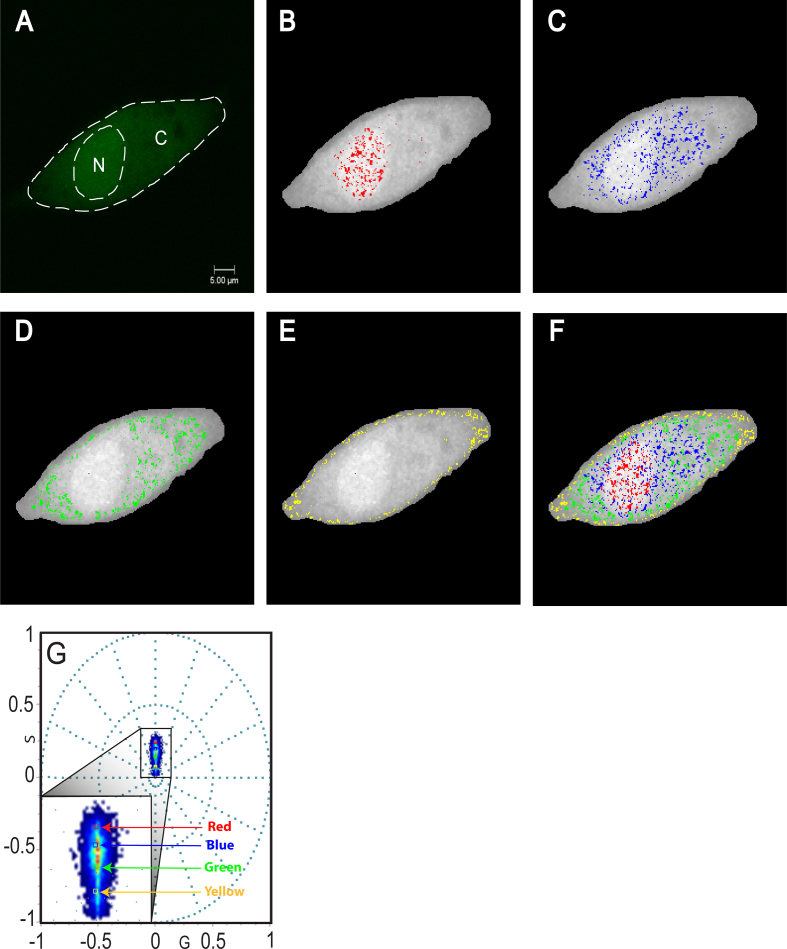


**figs9:**
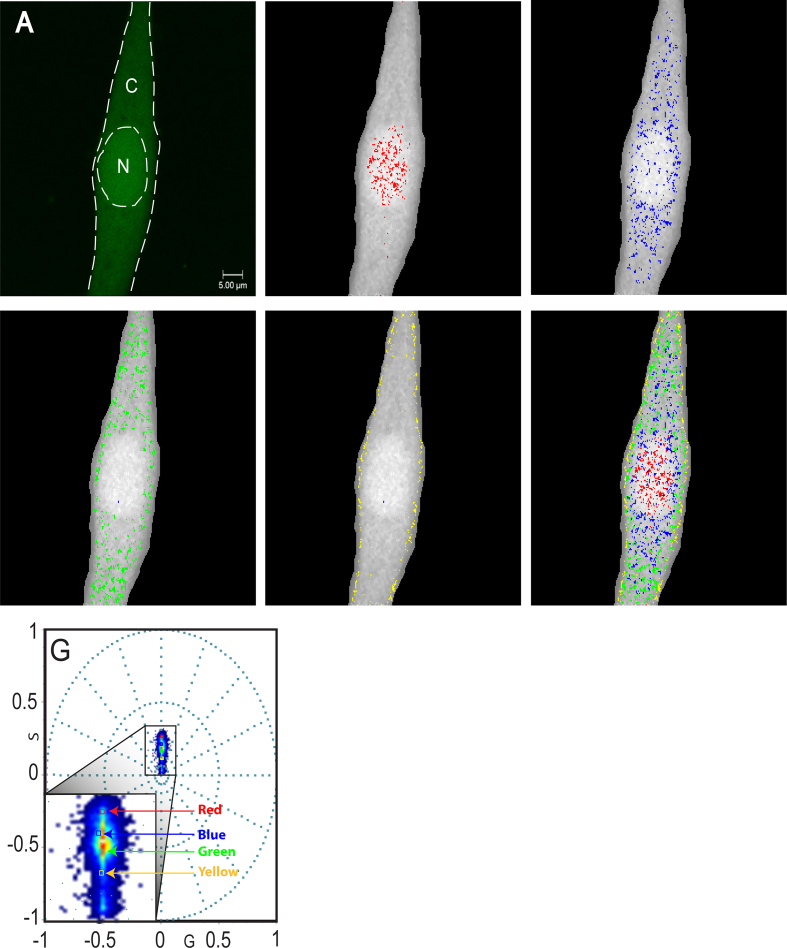


## Data Availability

Data will be made available on request.

## Abbreviations

FLIM: Fluorescence Lifetime Imaging
SPA: Spectral Phasor Analysis
*λ_max_*: Maximum Wavelength
Spectral Width: Full Width of the Half Maximum
OGB: Oregon Green™ BAPTA 1-AM
SR: Sarcoplasmic Reticulum
ROI: Region of Interest
