# Supplementary material for: Applying first & second harmonic spectral phasor analysis on a single-wavelength calcium fluorophore
Source: Biochem Biophys Rep. 2025 Feb 20;41:101956. doi: 10.1016/j.bbrep.2025.101956 (PMC11891743; doi:10.1016/j.bbrep.2025.101956)
Supplement: Multimedia component 1 [file mmc1.docx]

# **Supplementary Materials**

**S1 Fig. Conversion of LASX Files to Hyperspectral Readable Format.**

**(A)** SimFCS 4 is opened and the FLIM option is selected. **(B)** a menu is then opened and the lifetime form option is then selected. **(C)** the start and end wavelength are calibrated and **(D)** the Leica files are referenced into a hyperspectral readable format. **(E)** The converted file is then read and added to the software.

**S2 Fig. Masking Spectral Images to Remove Background**

**(A)** After the spectral file has been read into SimFCS 4, a spectral image is formed. The image is right-clicked and the Hand draw option is selected. **(B)** The Hand draw an enclosed area option is picked and then the user draws around the sample. The Click on area to fill option is selected, the user clicks on the centre of the drawn sample and the window is closed. **(C)** The Mask option is clicked active in the main menu and then the mask is applied to the sample.

**S3 Fig. Comparison of Fluorescence Images to Phasor Plots and Corresponding Spectral Images of Cells and Background Removed**

**(A)** Pseudo-brightfield of the cell set at 633 nm. **(B)** Fluorescence image with a 5µm scale bar, as well as labels for the cytoplasm (C) and nucleus (N). **(C)** The resulting phasor plot and corresponding spectral image following removal of the background using the masking tool. **(D)** The resulting phasor plot and corresponding spectral image of only the background, following the removal of the cell.

**S4 Fig. Replicate of Fig 1.**

**(A)** Fluorescence image with a 5µm scale bar, as well as labels for the cytoplasm (C) and nucleus (N). **(B)** first harmonic spectral phasor plot with a zoomed image showing all three cursors outlining the nucleus (red), cytoplasm (blue) and membrane (green). **(C)** spectral image that corresponds with the first harmonic phasor plot and each cursor applied. **(D)**. Average first harmonic λmax (left y-axis) and spectral width (right y-axis) of the nucleus (N), cytoplasm (C) and membrane (M) of all cells analysed. **(D)** Second harmonic spectral phasor plot with a zoomed image showing all three cursors outlining the nucleus (red), cytoplasm (blue) and membrane (green). **(E)** Spectral image that corresponds with the second harmonic phasor plot and each cursor applied. **(F)** Spectral image that corresponds with the second harmonic phasor plot and each cursor applied. **(G)** Average second harmonic λmax (left y-axis) and spectral width (right y-axis) of the nucleus (N), cytoplasm (C) and membrane (M) of all cells analysed.

**S5 Fig. Replicate of Fig 1.**

**(A)** Fluorescence image with a 5µm scale bar, as well as labels for the cytoplasm (C) and nucleus (N). **(B)** first harmonic spectral phasor plot with a zoomed image showing all three cursors outlining the nucleus (red), cytoplasm (blue) and membrane (green). **(C)** spectral image that corresponds with the first harmonic phasor plot and each cursor applied. **(D)**. Average first harmonic λmax (left y-axis) and spectral width (right y-axis) of the nucleus (N), cytoplasm (C) and membrane (M) of all cells analysed. **(D)** Second harmonic spectral phasor plot with a zoomed image showing all three cursors outlining the nucleus (red), cytoplasm (blue) and membrane (green). **(E)** Spectral image that corresponds with the second harmonic phasor plot and each cursor applied. **(F)** Spectral image that corresponds with the second harmonic phasor plot and each cursor applied. **(G)** Average second harmonic λmax (left y-axis) and spectral width (right y-axis) of the nucleus (N), cytoplasm (C) and membrane (M) of all cells analysed.

**S6 Fig. Replicate of Fig 2.**

**(A)** Fluorescence image with a 5µm scale bar, as well as labels for the cytoplasm (C) and nucleus (N). Individual spectral images were generated which highlight each region across the cell such as the **(B)** nucleus, **(C)** SR-like ROI enveloping and protruding from the nucleus, **(D)** cytoplasmic ROIs and **(E)** cell membrane. **(F)** An overlay of each cellular region highlighted by the cursors in **(G)** the spectral phasor plot was generated. **(H)** Average first harmonic λmax (left y-axis) and spectral width (right y-axis) of the nucleus (N), SR-like ROI (SR), cytoplasm (C) and membrane (M) of all cells analysed. Data is expressed as a column bar of the mean and SEM. Statistical analysis was conducted using the Repeated Measures ANOVA.

**S7 Fig. Replicate of Fig 2.**

**(A)** Fluorescence image with a 5µm scale bar, as well as labels for the cytoplasm (C) and nucleus (N). Individual spectral images were generated which highlight each region across the cell such as the **(B)** nucleus, **(C)** SR-like ROI enveloping and protruding from the nucleus, **(D)** cytoplasmic ROIs and **(E)** cell membrane. **(F)** An overlay of each cellular region highlighted by the cursors in **(G)** the spectral phasor plot was generated. **(H)** Average first harmonic λmax (left y-axis) and spectral width (right y-axis) of the nucleus (N), SR-like ROI (SR), cytoplasm (C) and membrane (M) of all cells analysed. Data is expressed as a column bar of the mean and SEM. Statistical analysis was conducted using the Repeated Measures ANOVA.

**S8 Fig. Replicate of Fig 3.**

**(A)** Fluorescence image with a 5µm scale bar, as well as labels for the cytoplasm (C) and nucleus (N). Individual spectral images were generated which highlight each region across the cell such as the **(B)** nucleus, **(C)** SR-like ROI enveloping and protruding from the nucleus, **(D)** cytoplasmic ROIs and **(E)** cell membrane. **(F)** An overlay of each cellular region highlighted by the cursors in **(G)** the spectral phasor plot was generated. **(H)** Average first harmonic λmax (left y-axis) and spectral width (right y-axis) of the nucleus (N), SR-like ROI (SR), cytoplasm (C) and membrane (M) of all cells analysed. Data is expressed as a column bar of the mean and SEM. Statistical analysis was conducted using the Repeated Measures ANOVA.

**S9 Fig, Replicate of Fig 3.**

**(A)** Fluorescence image with a 5µm scale bar, as well as labels for the cytoplasm (C) and nucleus (N). Individual spectral images were generated which highlight each region across the cell such as the **(B)** nucleus, **(C)** SR-like ROI enveloping and protruding from the nucleus, **(D)** cytoplasmic ROIs and **(E)** cell membrane. **(F)** An overlay of each cellular region highlighted by the cursors in **(G)** the spectral phasor plot was generated. **(H)** Average first harmonic λmax (left y-axis) and spectral width (right y-axis) of the nucleus (N), SR-like ROI (SR), cytoplasm (C) and membrane (M) of all cells analysed. Data is expressed as a column bar of the mean and SEM. Statistical analysis was conducted using the Repeated Measures ANOVA.

**S1 Table. First Harmonic λmax and Spectral Width Values (nm) in All Cells Analysed using Large Cursors (0.05).**

**S2 Table. Second Harmonic λmax and Spectral Width Values (nm) in All Cells Analysed using Large Cursors (0.05).**

**S3 Table. First Harmonic λmax and Spectral Width Values (nm) in All Cells Analysed using Small Cursors (0.005).**

**S4 Table. Second Harmonic λmax and Spectral Width Values (nm) in All Cells Analysed using Small Cursors (0.005).**
